# The Tetraspanin Tspan8 Associates with Endothelin Converting Enzyme ECE1 and Regulates Its Activity

**DOI:** 10.3390/cancers15194751

**Published:** 2023-09-27

**Authors:** Yingying Zhu, Julien Saint-Pol, Viet Nguyen, Eric Rubinstein, Claude Boucheix, Céline Greco

**Affiliations:** 1 UMR-S 1004, SFR André Lwoff, Inserm, 94800 Villejuif, France; julianzyy@hotmail.com (Y.Z.); julien.saintpol@univ-artois.fr (J.S.-P.); eric.rubinstein@inserm.fr (E.R.); 2 Université Paris Saclay, 91190 Gif-sur-Yvette, France; nguyviethu@hotmail.fr; 3 Department of Oncology, Tongji Hospital, Huazhong University of Science and Technology, Wuhan 430074, China; 4 Université Artois, UR 2465, Blood-Brain Barrier Laboratory (LBHE), 62300 Lens, France; 5 Plateforme Spectrométrie de Masse, Laboratoire Biochimie-Hormonologie, Hôpital Robert-Debré, Assistance Publique Hôpitaux de Paris (APHP), 75610 Paris, France; 6 Centre d’Immunologie et des Maladies Infectieuses, Inserm, CNRS, Sorbonne Université, CIMI-Paris, 75013 Paris, France; 7 UMR-S 1197, SFR André Lwoff, Inserm, 94800 Villejuif, France; 8 Department of Pain and Palliative Care, Hôpital Necker-Enfants Malades, Assistance Publique Hôpitaux de Paris (APHP), 75610 Paris, France; 9 U1163, IMAGINE Institute, Université de Paris Cité, Inserm, 75014 Paris, France

**Keywords:** Tspan8, endothelin, ECE1, colorectal carcinoma

## Abstract

**Simple Summary:**

Tetraspanins are cell surface proteins that induce the compartmentalization of molecules with receptor, signaling, or enzymatic properties in microdomains that could serve the integration of transmembrane signals. Several knockouts illustrate their biological functions such as infertility (CD9/CD81), lymphoma development (CD37), immunological deficiency (CD81), or renal insufficiency (CD151). The link between molecular interactions and organ defects remains often difficult to decipher, as is the relationship between the expression of some tetraspanins and the outcome of tumors. High expression of Tspan8 in tumors is associated with poor prognosis, which may be due to increased cell motility and angiogenesis. In a model of colorectal tumor, we explored Tspan8 interacting molecules by mass spectrometry and found that compared to CD9, Tspan8 specifically recruits the endothelin converting enzyme ECE1 to tetraspanin-enriched microdomains and positively modulates its enzymatic activity. Convergence of Tspan8 and endothelin axis in tumor biology offers a new area of research.

**Abstract:**

Tspan8 is a member of the tetraspanins family of cell surface molecules. The ability of tetraspanins to organize membrane microdomains with other membrane molecules and interfere with their function suggests that they could act as surface integrators of external or internal signals. Among the first identified tetraspanins, Tspan8 promotes tumor progression and metastasis, presumably by stimulating angiogenesis and cell motility. In patients, its expression on digestive tract tumors seems to be associated with a bad prognosis. We showed previously that Tspan8 associates with E-cadherin and EGFR and modulates their effects on cell motility. Using Mass spectrometry and western blot, we found a new partner, the endothelin converting enzyme ECE1, and showed that Tspan8 amplifies its activity of conversion of the endothelin-1 precursor bigET1 to endothelin. This was observed by transduction of the colon carcinoma cell line Isreco1, which does not express Tspan8, and on ileum tissue fragments of tspan8ko mice versus wild type mice. Given these results, Tspan8 appears to be a modulator of the endothelin axis, which could possibly be targeted in case of over-activity of endothelins in biological processes of tissues expressing Tspan8.

## 1. Introduction

Tspan8 (originally named Co-029 [1]) is a member of the tetraspanin family of cell surface proteins. Tetraspanins associate directly with other cell surface proteins forming primary complexes that combine together, presumably through tetraspanin-tetraspanin interactions, to build secondary complexes that link surface proteins of different properties (integrins, cytokine receptors, enzymes) in what is called tetraspanin enriched microdomains or tetraspanin web [2,3,4,5,6,7,8]. Secondary interactions are revealed by immunoprecipitation of Brij97 lysates. These complexes display dynamic changes presumably modifying cellular functions in relation to the microenvironment [9].

At the level of primary complexes, the consequences of tetraspanins interactions with their partners are diverse, comprising control of cell surface expression by cellular trafficking, receptor alteration of affinity for the substrate, and enzymatic regulation. Some specific partners for CD81, CD9, CD151 [8], CD37 [10], or TspanC8 (tetraspanins with eight cysteines in the large extracellular domain) [11] have already been reported. For Tspan8, crosslinking experiments suggest that E-cadherin might be a primary partner [12]. Tspan8 is mainly associated with epithelial cells of the digestive tract and its expression appears to be related to poor prognosis in gastrointestinal tumors and other types of cancers [13,14]. Modulation of E-cadherin/p120catenin expression leads to alterations of cell motility in Tspan8 expressing cells [12]. In an attempt to find other membrane specific partners of Tspan8, we performed MS analysis of Tspan8 precipitated protein complexes. Among many associated proteins [15], some of which, such as integrins, EGFR, or CD44, are classically found in tetraspanins complexes, we report a new protein partner, the endothelin converting enzyme 1 (ECE1).

ECE1 is one of the two endothelin converting enzymes that belong to the endothelin’s system, which includes endothelins and the endothelin G-protein-linked transmembrane receptors ETAR and ETBR. Endothelins form a family of three endogenous vasoactive 21 amino-acid-long peptides (ET1, ET2 and ET3) [16]. Endothelins have overlapping tissue distribution, with ET1 being the most widely expressed. ET1 is synthetized and released by epithelia, vascular smooth muscle cells, leukocytes and macrophages, cardiomyocytes, and endothelial and mesangial cells. It is characterized by a single alpha-helix and two disulphide bridges at Cys3-Cys11 and Cys1-Cys15. The human ET1 gene encodes the precursor pre-pro-ET1. Transcription of ET1 occurs in response to various stimuli, including thrombin, angiotensin II, and insulin but also physico-chemical stimuli and some drugs such as cyclosporine or cocaine [17]. Pre-pro ET1 is cleaved sequentially by different endopeptidases to form pro-ET1 and then a 38-amino-acid-long, biologically inactive precursor known as bigET1, which is further cleaved into ET1 by Endothelin Converting Enzymes 1–2 or alternatively by a chymase enzyme [18].

Endothelins have multiple activities: growth-promoting [19], cell migration and invasion [20], apoptosis [21], cell survival [22], vasoactive [23], and nociceptive properties [24,25,26] affecting the function of a number of tissues and systems. Their actions are primarily local, in a paracrine or autocrine manner. Elevated tissue or plasma concentrations of ET1 occur in a variety of pathological states, for example in metastasized prostate and breast cancer, following cutaneous injury or ischemic injury related to acute respiratory distress syndrome sepsis and disseminated intravascular coagulation, renal insufficiency, sickle cell disease, Raynaud’s disease, or cardiac insufficiency [24]. Experimental models suggest that the endothelin axis is implicated in the development and progression of cancer [27,28].

Following the discovery of the molecular association between ECE1 and Tspan8, we explored a functional relationship between these two molecules since, as mentioned above, there are many situations in which tetraspanins affect the function of their molecular partners. We mainly analyzed the effect of Tspan8 on bigET1 conversion to ET1 by ECE1.

## 2. Materials and Methods

### 2.1. Cell Lines

The cell line Isreco1 was initially derived from a primary human colon cancer (Duke’s C, class III) surgical specimen [29]. Isreco1 cells were transduced to express the tetraspanin Tspan8 and were called Is1-tspan8 [12]. Phenotypic characteristics of the Isreco cell lines (morphology and surface markers) were reported previously [6]; they are checked after each thawing and before a set of experiments in order to avoid contamination between cell lines. Sequenom analysis with the MassARRAY system showed that Isreco1 and Tspan8 transduced cells harboured a G12D homozygous mutation of KRAS, whereas no mutations of BRAF and PI3K were found. The cell lines were further characterized by transcriptomal analysis (Miltenyi Biotech Microarray Service, Bergisch Gladbach, Germany) using Agilent Whole Genome Oligo Microarrays (4 × 44 K one color) (Agilent, Santa Clara, CA, USA).

### 2.2. Antibodies

The anti-tetraspanin mAb TS9 against CD9, TS29.1 (IgG1) and TS29.2 (IgG2b) directed against Tspan8, used in this study were produced in our laboratory [12]. The following commercial antibodies were purchased: anti human ECE1 (Goat IgG, R & D System) and biotinylated anti human ECE1 (Goat IgG, R & D System) and used according to manufacturer’s instructions. Anti-tetraspanin antibodies were used as primary antibodies for immunoblotting (1 μg/mL), flow cytometry (10 μg/mL, 50 μL for 0.2 to 1 × 10^6^ cells), and in-situ labelling (10 μg/mL). For functional studies, 20 μg/mL of mouse intestinal ileum fragments was added to the incubation medium.

For production of rat mAbs to mouse Tspan8 (mtspan8), an expression vector containing the coding region of mouse tspan8 fused to GFP was transfected in Hek cells. Positive cells were selected by 3 cycles of cell sorting. The presence of a fusion protein of the appropriate size was checked by western blot with antibodies to GFP protein. Two rats were injected with 10^7^ cells at days 1, 8, and21. They were boosted twice at days 29 and 37 with a GFPtrap precipitation of a Brij97 lysate of 10^8^ Hek-mtspan8/GFP cells and one rat was sacrificed 4 days after the last injection to recover spleen lymphocytes, which were then fused to ×63 cells. Hybridoma supernatants were screened by flow cytometry on a mixture of Hek wild type cells and Hek-mtspan8/GFP with an Alexa Fluor 647 anti-rat antibody. Antibodies specific for Hek-tspan8/GFP were further characterized by western blot on Hek cells transfected with mouse mtspan8 and on mouse digestive tissues. A single fusion was sufficient for obtaining eight hybridomas secreting antibodies that appeared to be specific. Two antibodies, Z14 and Z17, were retained for further studies. All tetraspanins monoclonal antibodies, which recognize their target by membrane immunofluorescence on living cells, are directed towards the large extracellular domain.

### 2.3. Production of Tspan8 Knockout Mice

Tspan8 knockout (tspan8ko) mice were produced at Institut Clinique de la Souris (ICS, Strasbourg, France). The targeting strategy was to delete exon 1–2 and upstream promoter region and downstream intron 2 (1.8 kb) using the flox strategy (Supplementary Figure S1). For that purpose, a targeting vector containing 5′ and 3′ homology arms of 4.5 kb and 3.5 kb, with a replacement sequence containing a 5′ genomic fragment with 1.3 kb promoter, 379 bp corresponding to exons 1 and 2 separated by intron 1 (coding sequence starts in exon 2) and 0.25 kb to intron 2 followed by 3′ PGK-Neo resistance genes flanked by FRT sites. The whole replacement sequence was flanked by lox sites. Sequence data are indicated according to Genome Data Viewer https://www.ncbi.nlm.nih.gov/genome/gdv/ accessed on 1 May 2023, (NC_000076.7 of GRCm39 assembly). After recombination in the ES cell line BD10 [ICS-C57BL/6NTac], selection, cloning, and control of the recombination by southern blot, the PGK-Neo cassette was excised by transfection with an expression vector encoding FLT, and one clone of ES cells (K656BD10–16) was introduced into blastocysts. F1 mice were crossed with Cre mice to remove the promoter-exon1-intron sequence to generate knockout mice. Knockout mice were transferred to our laboratory for in-house breeding and experimental use. Tspan8 knockout mice were kept on a C57BL/6N background. Wild type control mice and homozygous tspan8 knockout mice originated from the same breeding. The absence of protein expression was checked using rat monoclonal antibodies produced against mouse Tspan8.

### 2.4. Endothelin Dosage

For endothelin-1 dosage, we used the QuantiGlo Human Endothelin-1 Chemiluminescent Immunoassay (R & D System) as specified by the manufacturer. Cells were seeded at 3 × 10^5^ cells/well in DMEM with 10% FCS in 24 well microplates and bigET1 was added after 24 h and left for 5 to 24 h. Supernatants were then harvested for ET1 dosage. All tests were performed in duplicates. The kit also detects mouse ET1.

### 2.5. Preparation of Intestinal Tissues and DNA Dosage

Intestinal pieces were extracted from the ileum of wt and tspan8ko mice sacrificed by cervical dislocation. Small intestines (SI) were washed in ice-cold PBS and sliced longitudinally to optimize the wash. SI were then rinsed 3 × 20 min in ice-cold PBS at 4 °C under slight agitation. Ileum regions were cut into small pieces (0.8 mm^−1^ cm long) and put in culture in μ-Slide 8 well (ibidi, Clinisciences, Nanterre, France) overnight with 250 μL of DMEM/Ham’s F-12 Glutamax supplemented by 2% (*v/v*) penicillin/streptomycin/neomycin, 1 × B27 (Life Technologies SAS, Courtaboeuf, France), 100 ng/mL noggin, 500 ng/mL R-spondin1, 20 ng/mL EGF, and 10 ng/mL m-bFGF. Then, 100 ng/mL of bigET1 or vehicle were added into the wells to study the conversion of bigET1 into ET1 and the endogenous production of ET1, respectively. The amount of ET1 converted or endogenously produced was dosed by ELISA and reported by ng of gDNA (dosed from the ileum pieces).

For DNA extraction, we used the PureLink^®^ Genomic DNA Kit (Life Technologies SAS) according to manufacturer’s protocol. Tissues were lysed for 4 h, vortexed 10–15 s, then a double elution was performed to increase gDNA yield. DNA content was measured with the Qubit^®^ 3.0 Fluorometer (Life Technologies SAS).

For statistical analysis of converted endothelin, data were processed in Prism Graphpad 9.5.1 as Column analysis (2 columns with 2 sub-columns), with the following parameters recommended by the software: nonparametric test, experimental design: paired. The Wilcoxon matched pairs rank test was selected. Options: two-tail recommended. For comparison of antibodies effects on bigET1 conversion, the number of pairs was too low for the Wilcoxon test and only the 2-way ANOVA test was used.

### 2.6. Immunofluorescence

For flow cytometry analysis of surface molecules, cells were detached using a non-enzymatic solution (Life Technologies SAS). For in-situ labelling, frozen sections of tissues were prepared and fixed with −20 °C acetone for 5′.

### 2.7. Immunoprecipitation (IP) and Biotinylation of Cell Surface Proteins

Biotin-labelling of surface proteins was performed with 0.5 mg/mL EZ-link-Sulfo-NHS-LC-biotin (Life Technologies SAS) in PBS buffer. After 3 washings in PBS, the cells were lysed in a lysis buffer (30 mM Tris pH 7.4, 150 mM NaCl, 1 mM CaCl_2_, 1 mM MgCl_2_, 1 mM phenylmethylsulfonyl fluoride, 0.5 mg/mL leupeptin, 1 mg/mL pepstatin A, and 10 kallikrein-inactivating units/mL aprotinin) supplemented with 1% Brij 97 (Roche Molecular Biochemicals, Meylan, France). After 30 min incubation at 4 °C, the insoluble material was removed by centrifugation for 15 min at 10,000× *g* and the cell lysate was precleared by addition of heat inactivated goat serum and protein G sepharose beads (Dutscher, Issy-les-Moulineaux, France). Proteins were then immunoprecipitated by adding 1 μg mAb and 20 μL protein G-sepharose beads to 200–400 μL of the lysate. The sepharose beads were washed in the Brij97 lysis buffer. Half were directly boiled in Laemmli buffer. The other half was eluted in 1 mL Triton ×100 at 1% in the lysis buffer without CaCl_2_/MgCl_2_. The eluate was then precipitated with 25 μL/mL streptavidin beads (Thermofisher Scientific) to recover biotinylated membrane proteins, washed with the Tris-HCl/Triton ×100 buffer, and boiled in Laemmli buffer containing 3 mg/mL free biotin. When appropriate, 2% β-mercapto-ethanol was added to Laemmli buffer. The immunoprecipitated proteins were separated by SDS-polyacrylamide gel electrophoresis, transferred to a PVDF membrane (Dutscher), and visualized using the Odyssey Infrared Imaging System (LI-COR Biosciences, Bad Homburg, Germany) after incubation of the membrane with Alexa Fluor 680-labelled streptavidin (Life Technologies SAS).

### 2.8. Mass Spectrometry Protein Identifications [15]

Immunoisolation of Tspan8-containing or CD9-containing molecular complexes was obtained by in situ lysis of Isreco1 (Tspan8 negative) and Is1-tspan8 cell lines in the mild detergent Brij97. After removal of insoluble material by centrifugation and preclearing with NHS-activated Sepharose™ High Performance, GE Healthcare beads coupled to BSA and to goat serum (Sigma, Saint-Quentin-Fallavier, France), isolation of Tspan8 and CD9 containing complexes was performed using beads coupled to mAb TS29.2 or TS9, respectively. Beads were washed and eluted by boiling for 3 min in Laemmli buffer containing 1% SDS. After separation by 5–15% SDS-polyacrylamide gel electrophoresis under non-reducing conditions, gel fragments were excised and digested by trypsin. Following reduction and alkylation, peptides were identified by mass spectrometry (LTQ-Orbitrap Velos, Thermo Fisher Scientific, Waltham, MA, USA)) coupled online with a nano-HPLC system Dionex Ultimate 3000Thermo Fisher Scientific, Waltham, MA, USA).

The data were analyzed by the Proteome Discoverer 1.4 software. The database is human (Swiss-Prot).

A relative quantitation was performed with the Proteome Discoverer integrated label free method, which consists of comparing the mean peaks area of the three best peptides to a given protein from one sample to another. The method of calculation is three dimensional, relying on retention time, ion intensity, and *m/z* ratio of the peptide, with a mass error lower than 2 ppm.

## 3. Results

### 3.1. Tspan8 Molecular Partners

**Figure 1:**
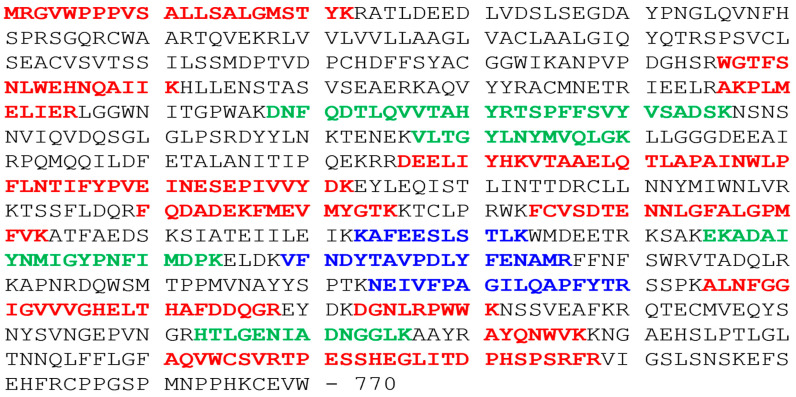
Endothelin-converting enzyme 1 OS = Homo sapiens GN = ECE1 [ECE1_HUMAN] Isoform ECE1-1b—Aminoacid sequences of ECE1 peptides identified by MS in TS29.2 coprecipitates from Is1-Tspan8 cells. Peptide identifications were validated by determination of false positives by Target decoy PSM validator. It is high (green) if the false positive rate (FDR or false Discovery rate) is less than 1%, low (red) if the FDR is greater than 5% and average (blue) (medium between 1 and 5%).

In order to precisely identify Tspan8 molecular connections, we have used mass spectrometry to identify molecules that could be closely associated to Tspan8 within the tetraspanin web. We have analyzed CD9 and Tspan8 immunoprecipitates of the cell lines Isreco1 and Is1-tspan8 after lysis in the mild detergent Brij97 and SDS-Page separation. The Tspan8 immunoprecipitate of Isreco1 served as negative control and allowed us to identify and exclude proteins that were not specifically precipitated under our experimental conditions. Under these conditions, we obtained >1000 co-immunoprecipitated proteins; by comparison with Swissprot database, it appeared that 300 were plasma cell membrane related. Fifty-six plasma membrane proteins were found associated with both CD9 and Tspan8 in Is1-tspan8 cells and with CD9 in Isreco1. Details of these results were reported in Zhu et al. [15]. We now report that among plasma cell membrane proteins, ECE1 and Tspan6 were co-immunoprecipitated with Tspan8 and with CD9 mAbs only in Is1-Tspan8 cells and not with CD9 mAb in Isreco1 cells (Table 1). Three and seven ECE1 unique peptides (high and medium validated peptides by target decoy PSM validator, shown in Figure 1) were found in the CD9 and Tspan8 immunoprecipitates of Is1-Tspan8 extracts, respectively, whereas no ECE1 peptides were found in Isreco1 extracts immunoprecipitated by CD9 or Tspan8 antibodies.

### 3.2. Association of ECE1 with Tspan8 Is Confirmed by Western Blot

**Figure 2:**
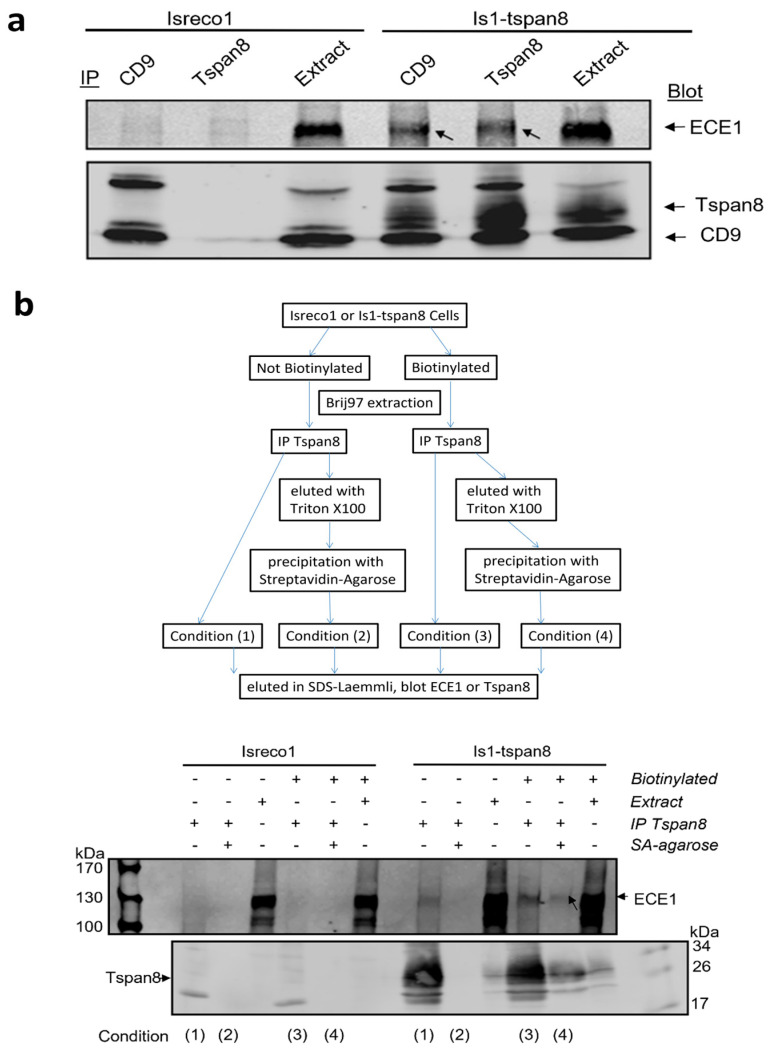
Western blots of Tspan8 (TS29.2) or CD9 (TS9) coprecipitations. (**a**) Neither TS9 nor TS29.2 coprecipitated ECE1 in Isreco1 cells, whereas ECE1 was coprecipitated by both mAbs in Is1-tspan8 cells. (**b**) Upper panel: strategy for demonstrating that the association of Tspan8 and ECE1 occurs at cell surface. Lower panel: the coprecipitation of the 2 biotinylated surface proteins is observed in condition 4 in Is1-tspan8 cells.

As shown in Figure 2, we confirmed this association by western blot since ECE1 could be detected in both the CD9 and Tspan8 immunoprecipitates collected from Brij97 lysates of Is1-tspan8 cells but not in immunoprecipitates of parental Isreco1 cells. Because none of the commercial antibodies to ECE-1 was able to IP ECE1, the reverse demonstration of co-immunoprecipitation of tetraspanins by ECE1 could not be performed.

### 3.3. ECE1 Is Associated with Tspan8 at the Plasma Membrane

The available antibodies did not allow membrane detection of ECE1 by immunofluorescence. To demonstrate that the association between Tspan8 and ECE1 occurs at the cell surface, we biotinylated the cell surface proteins before lysis using Brij97 detergent to preserve the interactions and immunoprecipitation of Tspan8 with the mAb TS29.2. After washing in Brij97 buffer, half of the proteins were eluted in Laemmli buffer (with or without 2% β-mercapto-ethanol). The other half was eluted with Triton ×100 in order to dissociate the tetraspanin complexes. The biotin-labelled proteins were isolated using streptavidin coated beads and eluted in Laemmli buffer (with or without 2% β-mercapto-ethanol) supplemented with an excess of biotin (3 mg/mL) in order to avoid reassociation of biotinylated proteins to the beads. The gel electrophoresis was performed under non-reducing conditions for immunoblot with TS29.2 and reducing conditions for immunoblot with ECE1 antibody. Figure 2b shows that a band co-migrating with ECE-1 was detected in the Tspan8 immunoprecipitate in approximately the same proportions as Tspan8, whether the proteins immunoprecipitated with Tspan8 were isolated in a second step with streptavidin (thus present on the cell surface, condition 4) or not (condition 3). This band is specific since it is detected neither in the immunoprecipitates collected from Isreco1 lysates nor in those collected from non-biotinylated samples after streptavidin isolation (condition 2). We therefore conclude that the interaction between Tspan8 and ECE-1 occurs at the cell surface.

### 3.4. Modulation of bigET1 to Endothelin Conversion by Cellular ECE1 in the Presence of Tspan8

**Figure 3:**
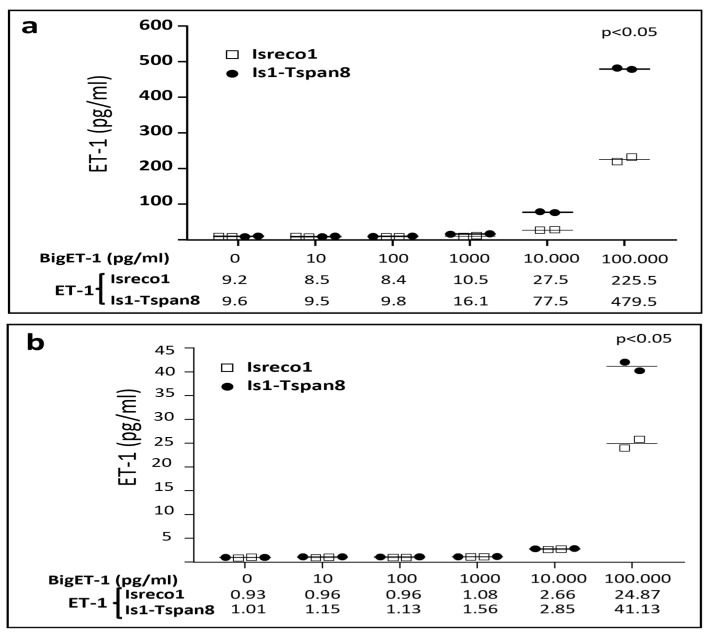
Conversion of bigET1 by Isreco1 and Is1-Tspan8 cells. (**a**) Increasing concentrations of bigET1 were added to the supernatants of Isreco1 and Is1-Tspan8 cells. A basal concentration of ET-1 of around ~9 pg/mL was found in the supernatant of the cells after 24 h culture in DMEM-FCS 10% medium. The superior conversion by Is1-Tspan8 cells appears at the concentration of 1000 pg/mL bigET1. It is assumed that less than 1% of added synthetic bigET1 can be converted to ET1 by the cells. (**b**) Cells were kept for 5 h in FCS free medium (they start to die if the incubation is longer). A difference of bigET1 conversion is still observed in confluent cells. (Wilcoxon matched-pairs signed rank test).

**Figure 4:**
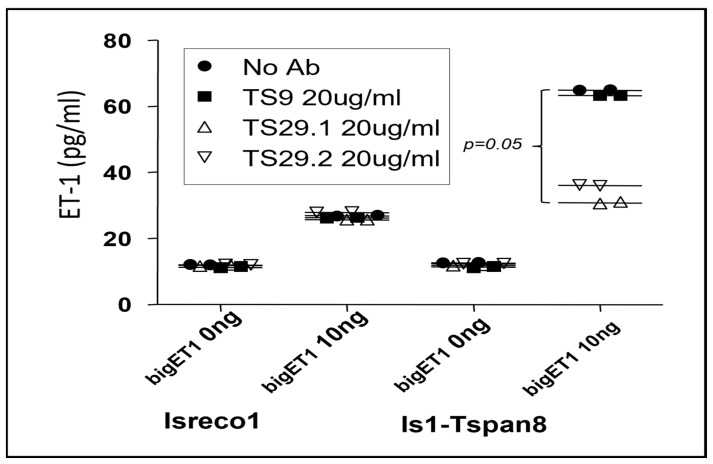
Effect of antibodies to CD9 (TS9) and Tspan8 (TS29.1, TS29.2) on conversion of bigET1 (10 ng) to ET1 in cells negative or positive for Tspan8 expression. Both cell lines can convert bigET1 into ET1 but this property is increased in Is1-Tspan8 cells, however the 2 Tspan8 antibodies TS29.1 and TS29.2 neutralize this property whereas the CD9 antibody has no effect. There is no effect of Tspan8 antibodies on the basal secretion of ET1 (bigET1 0 ng). (Prism graphpad, two-way anova).

In order to determine if the association between Tspan8 and ECE1 was of functional relevance, we analyzed the conversion of bigET1 into ET1 by Isreco1 cells expressing Tspan8 or not. In the supernatants of both parental and Tspan8 transfected cells, at confluence for 24 h, we observed a basal level of 10 pg/mL. No ET1 could be measured in the culture medium in the absence of cells. BigET1 was added into the culture medium at concentrations ranging from 10 pg/mL to 100 ng/mL before measurement of ET1 concentration after 24 h incubation. There was a differential efficiency of conversion according to the expression of Tspan8 that appeared at 1000 pg/mL of bigET1 (Figure 3A). At 10 ng/mL and 100 ng/mL bigET1, there was a ~2.5-fold increase in the concentration of ET1 in Isreco-1/Tspan8 cells when compared to parental Isreco1 cells. In these experiments, the maximal conversion of bigET1 to ET1 did not exceed approximately 0.5%. One explanation could be that natural bigET1 is a complex synthetic peptide with S-S bonds that may be difficult to reconstitute by chemical synthesis and that only a fraction of the peptide has the appropriate structure for being cleaved by ECE1. The increase in ET1 production observed in Isreco1/Tspan8 cells was prevented by the two anti-Tspan8 mAbs, TS29.1 and TS29.2, but not the anti CD9 mAb TS9 (Figure 4).

The experiments were repeated in FCS free medium for a shorter duration since the cells do not behave well in these conditions. If the level of conversion was reduced, however, a similar difference was observed between Isreco1 and Is1-tspan8 cells at high Big-ET1 concentrations (Figure 3B).

### 3.5. Tspan8 Regulation of BigET1 Conversion by Intestinal Tissue

**Figure 5:**
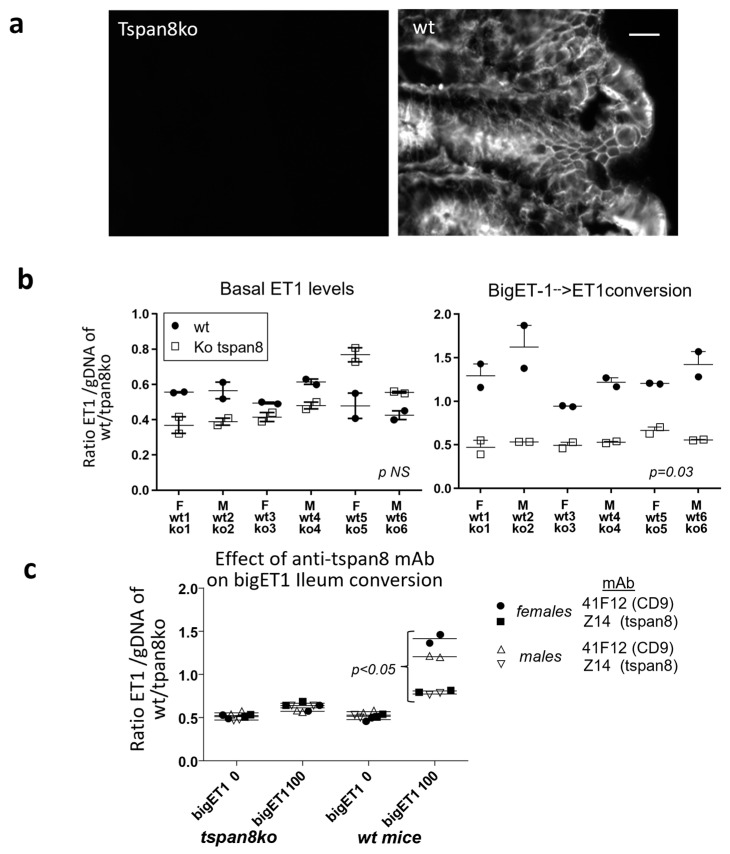
Secretion of ET1 and conversion of bigET1 by ileum fragments of wt versus tspan8 knockout mice, (**a**) The immunofluorescent labelling of colonic mucosa with the tspan8 rat monoclonal antibody Z17 directed towards mouse tspan8 shows the absence of expression in sections from the knockout mice (**left**) whereas the epithelium is strongly labelled in the wild type mouse (**right**). Scale bar 20 μm, same exposure time. (F = female, M = male) (**b**) Ileum sections of wt and Tpans8ko mice were incubated for 20 h in the presence (**right**) or absence (**left**) of bigET1 (100 ng/mL). Results are expressed as the ratio of ET1/.5 mL to 1 μg genomicDNA (gDNA) in wt and tspan8ko mice. (Prism graphpad, Wilcoxon matched-pairs signed rank test). (**c**) The ability of wt ileum to convert bigET1 (100 ng/mL) to ET1 is reduced by anti-mouse Tspan8 mAb compared to anti-mouse CD9 mAb. (Prism graphpad, Two-way Anova).

We then wanted to test the ability of Tspan8 to regulate BigET1 conversion in a more physiological model. Because Tspan8 is mainly expressed by intestinal epithelial cells (Figure 5A), we analyzed the endogenous secretion of endothelin-1 and BigET1 conversion by wt and tspan8ko intestinal fragments ex vivo. To normalize ET1 measurement to the number of cells in these fragments, we extracted and quantified the gDNA of the whole fragment at the end of the incubation time. The amount of ET1 (pg/mL) per 1μg of genomic DNA (ET1/gDNA) in the sample was then calculated and the results are presented as a ratio (ET1/gDNA). There was no difference in the amount of ET1 in the medium of wt and tspan8ko intestines. In contrast, a significant difference of bigET1 conversion could be detected in wt tissues, showing 1.9 to 3× more efficiency than tspan8ko tissues (Figure 5b). This effect in wt intestinal tissues is partially reverted by anti-mstpan8 Z14 rat mAb; the anti-mouse CD9 rat mAb 4.1F12 rat mAb had no effect (Figure 5c). These antibodies were also inactive in tspan8ko tissues and on conversion of endogenous secretion of ET1. In other terms, antibodies to human or mouse Tspan8, on human tumor cells (Figure 4) or normal mouse intestinal tissues, respectively, inhibit specifically a functional effect of Tspan8 that results in the increase of ECE1 activity.

## 4. Discussion

Tspan8 has a restricted pattern of expression. It is found mainly on epithelial cells of the digestive tract. It has attracted interest because, when expressed on tumor cells, it regulates cell motility and survival and is involved in the promotion of angiogenesis [30,31]. At the molecular level, Tspan8 promotes invasion by ProMMP9 activation or β-catenin loop stimulation in melanoma [32], regulates E-cadherin/catenin signalling and metastasis in colorectal [12] and breast cancer [33], and induces cancer stemness through sonic hedgehog signalling in breast cancer [34]. The expression of Tspan8 in tumor tissues has been shown to be regulated by multiple transcription factors or mediators such as LMCR1 and p53 in melanoma [35,36], SOX9 in pancreatic cancer [37], by long non coding RNA SOX2-AS1 in lung adnocarcinoma [38], and by Lysine Specific Demethylase 1 (LDS1) in CRC [39].

In an attempt to look for partners of Tspan8 that could contribute to its cellular effects, we compared, the tetraspanins complexes by MS in the cell line Isreco1 and the corresponding Tspan8 transduced Is1-Tspan8 cell line. Many proteins associated with tetraspanin complexes were found after immunoprecipitation with CD9 or Tspan8 antibodies. Isreco1 immunoprecipitation by TS29.2 was used as negative control to discard nonspecifically precipitated proteins. Interestingly, only two proteins, tspan6 and ECE1, were found to be associated with tetraspanin complexes when only Tspan8 was expressed. The level of ECE1, estimated by label free quantification was higher than tspan6 and had the advantage of having a function clearly identified. ECE-1 is a large dimeric molecule with four isoforms differing in their N-terminal domain, resulting in molecules varying from 754 to 770 a.a. It is a member of the M13 family of zinc metalloproteases that includes neprilysin, or NEP, to which ECE-1 has 37% identity [40]. It forms homodimers in which the two partners are linked by a disulfide bond.

Since the endothelin axis has been associated with protumoral properties in various models of tumors [27], we explored the characteristics of Tspan8 association to ECE1. We could confirm its association by western blot and demonstrated that it occurred at the surface of the cells. We then measured the ability of the cells to convert bigET1 to ET1, which showed a three fold increase of the conversion when Tspan8 was expressed. The increase of ET1 conversion in Tspan8 expressing tissues was confirmed by comparing the conversion of bigET1 by ileum fragments of wild type and tspan8ko mice kept in culture for 24 h.

The endothelins are extremely powerful peptides that require a precise regulation of the different component of the endothelin system, secretion of mature endothelin controlled at the level of transcription, and maturation of the precursors with the last step being the bigET1 conversion by ECE enzymes. The level of expression of receptors, their affinity, and their tissue distribution are the other aspect of the regulation of the activity of endothelins. ETBR appears to bind the three endothelins with similar affinity, whereas the affinity of ET1 and ET2 for ETAR is much higher than E3.

ECE1 is widely expressed [41,42] and is found on the cell surface as well as intracellularly and cleaves big-ETs in both locations [43,44] whereas ECE2 is found intracellularly and active at pH5.8 [45]. It is suggested that ET1 acts mainly locally as a paracrine mediator since it has a very short plasmatic half-life (below 5 min). Endothelins have roles in the control of numerous factors in cancer development and progression by acting either directly on tumor cells or through stromal components and targeting angiogenesis, stromal reaction, epithelial mesenchymal transitions, apoptosis, invasion, metastasis, and drug resistance [27]. The expression of the different components of the endothelin system has been investigated in cancer cell lines. However, there are only a few reports related to ECE in cancer. In the ovarian cell lines, OVCAR3 and ES2 expressing different components of the ET1 system (ET1, ECE-1 and ETAR), ECE1 RNAi inhibited ET1 secretion, reduced invasiveness, and increased adherence to extracellular matrix proteins and E-cadherin expression. In the oral squamous carcinoma cell line that also expresses the ET1 system components ET1, ETAR, and ETBR, as well as ECE-1, ET1 increases cell proliferation whereas antagonists of the receptors or ECE-1 RNAi reduced cell proliferation [46].

Egidy et al. [47] performed in situ localization of ET1 (large precursor polypeptide PPET-1), ECE1, and ETAR/ETBR in normal colon, adenoma, and carcinoma ce;;s. They showed that in colon carcinoma and normal colon epithelial cells, PPET-1 and ECE-1 were found mainly in epithelial and endothelial cells, whereas ETAR was found in smooth muscle cells and in cancer associated fibroblasts. ETBR was abundant in cancer stroma, whereas it was nearly undetectable in the normal colonic mucosa. These data suggest that in the colon, the production of ET1 is dependent on epithelial and endothelial cells whereas the activity of ET1 is directed towards stromal components, which may differ between normal and malignant tissues. This points towards the endothelin axis as a modulator of tumor microenvironment by tumor cells. Concerning angiogenesis, a reduction of vascular sprouting in aortic ring assay was reported in a model of tspan8ko mice and an even greater effect was observed in Tspan8/CD151 double knock out mice [48]. We could not observe this effect in our model of tspan8ko mice.

The expression of the ET1 axis components in the work of Egidy et al. corresponds to our results in the cell lines Isreco1 and Is1-Tspan8 that express ECE-1 and produce ET1 but do not express ET1 receptors at mRNA levels.

The way Tspan8 may regulate the properties of ECE1 remains to be determined. Even if the amount of total ECE1 is the same in Isreco1 and Is1-Tspan8 cells, an effect on molecular traffic cannot be excluded. A direct effect on enzyme activity could be linked to a change of affinity of ECE1 for its ligand. There are various examples of tetraspanins changing the ligand binding activity of a receptor for its ligand; for instance, laminin binding to alpha6-beta1 integrin is increased by CD151 [49], diphtheria toxin binding to the membrane precursor of HB-EGF depends on CD9 [50], and CD81 increases the avidity of α4β1 for VCAM-1 [51]. Another possibility is the activation of ECE1, which relies on the phosphorylation of its cytoplasmic region.

Independently of the mechanism of action, the biological significance of this phenomenon needs to be addressed. tspan8ko mice could be an appropriate tool for evaluating this question. It must be noticed that the absence of Tspan8 did not change the basal level of ET1, but the effect of Tspan8 is detectable when there is an exogenous supply of the ECE1 substrate biET1. This suggests that the enzymatic activity is reduced in tspan8ko mice and cannot cope easily with an increase of bigET1 availability. Therefore, situations where the ET1 axis is stimulated, such as pain or smooth muscle contraction [26], should be analyzed. In these situations, it would be of interest to see if targeting Tspan8 could be a useful method for reducing the activation of the endothelin axis.

## 5. Conclusions

We have demonstrated that the Tetraspanin Tspan8 recruits ECE1 to tetraspanin’s molecular domains at the cell surface. In addition, when Tspan8 is expressed, the conversion of BigET1 to endothelin-1 is increased. Since both the endothelin system and Tspan8 may favor tumor growth, this work opens the way for further investigation of potential targets for interfering with a synergistic effect that may be deleterious in the development of cancer. In this regard, antibodies to Tspan8 reverse the effect of Tspan8 expression on conversion of BigET1 to endothelin by ECE1.

## Figures and Tables

**Table 1:** Tspan8 is required for the association of ECE1 and Tspan6 to tetraspanin protein complexes. MS identification of plasma membrane proteins associated with CD9 and Tspan8 complexes after IP by TS9 (CD9) or TS29.2 (Tspan8) mAbs of Brij97 extracts from Isreco1 and Is1-tspan8 cells. ECE1 and Tspan8 (bold characters) were found in the tetraspanin complexes of Is1-tspan8 cells after CD9 or Tspan8 immunoprecipitation (IP) but not in Isreco1 CD9 IP. S-area are values obtained by label free quantification of identified proteins.As an indication, the quantification of ITGB1 and NOTCH2 which are the non tetraspanin proteins associated with respectively the highest and lowest S-area values in the Brij97 tetraspanin protein complexes.

Proteins (Genes Names)	Isreco1—IP CD9			Is1-tspan8—IP CD9			Is1-tspan 8—IP Tspan8		
	Peptides (n)		S-Area	Peptides (n)		S-Area	Peptides (n)		S-Area
	Total	Unique		Total	Unique		Total	Unique	
**ECE1**	**0**	**0**	**0**	**10**	**3**	**2.62 × 10^7^**	**15**	**7**	**1.13 × 10^8^**
**Tspan6**	**0**	**0**	**0**	**3**	**1**	**3.64 × 10^7^**	**4**	**2**	**2.56 × 10^7^**
ITGB1	29	18	2.72 × 10^9^	28	17	3.03 × 10^9^	27	16	2.43 × 10^9^
NOTCH2	5	1	7.98 × 10^6^	3	1	1.30 × 10^7^	5	3	5.07 × 10^6^

## Data Availability

The data presented in this study are available in this article.

## Supplementary Materials

The following supporting information can be downloaded at: https://www.mdpi.com/article/10.3390/cancers15194751/s1, Figure S1: Strategy for homologous recombination of the Tspan8 gene.

## References

[1] Szala S., Kasai Y., Steplewski Z., Rodeck U., Koprowski H., Linnenbach A.J. (1990). Molecular cloning of cDNA for the human tumor-associated antigen CO-029 and identification of related transmembrane antigens. Proc. Natl. Acad. Sci. USA.

[2] Rubinstein E., Le Naour F., Lagaudriere-Gesbert C., Billard M., Conjeaud H., Boucheix C. (1996). CD9, CD63, CD81, and CD82 are components of a surface tetraspan network connected to HLA-DR and VLA integrins. Eur. J. Immunol..

[3] Boucheix C., Rubinstein E. (2001). Tetraspanins. Cell. Mol. Life Sci..

[4] Berditchevski F. (2001). Complexes of tetraspanins with integrins: More than meets the eye. J. Cell Sci..

[5] Hemler M.E. (2005). Tetraspanin functions and associated microdomains. Nat. Rev. Mol. Cell Biol..

[6] Le Naour F., Andre M., Greco C., Billard M., Sordat B., Emile J.F., Lanza F., Boucheix C., Rubinstein E. (2006). Profiling of the tetraspanin web of human colon cancer cells. Mol. Cell. Proteom..

[7] Charrin S., Le Naour F., Silvie O., Milhiet P.E., Boucheix C., Rubinstein E. (2009). Lateral organization of membrane proteins: Tetraspanins spin their web. Biochem. J..

[8] Charrin S., Jouannet S., Boucheix C., Rubinstein E. (2014). Tetraspanins at a glance. J. Cell Sci..

[9] Espenel C., Margeat E., Dosset P., Arduise C., Le Grimellec C., Royer C.A., Boucheix C., Rubinstein E., Milhiet P.E. (2008). Single-molecule analysis of CD9 dynamics and partitioning reveals multiple modes of interaction in the tetraspanin web. J. Cell Biol..

[10] Peeters R., Cuenca-Escalona J., Zaal E.A., Hoekstra A.T., Balvert A.C.G., Vidal-Manrique M., Blomberg N., van Deventer S.J., Stienstra R., Jellusova J. (2022). Fatty acid metabolism in aggressive B-cell lymphoma is inhibited by tetraspanin CD37. Nat. Commun..

[11] Jouannet S., Saint-Pol J., Fernandez L., Nguyen V., Charrin S., Boucheix C., Brou C., Milhiet P.E., Rubinstein E. (2015). TspanC8 tetraspanins differentially regulate the cleavage of ADAM10 substrates, Notch activation and ADAM10 membrane compartmentalization. Cell. Mol. Life Sci..

[12] Greco C., Bralet M.P., Ailane N., Dubart-Kupperschmitt A., Rubinstein E., Le Naour F., Boucheix C. (2010). E-cadherin/p120-catenin and tetraspanin Co-029 cooperate for cell motility control in human colon carcinoma. Cancer Res..

[13] Bonnet M., Maisonial-Besset A., Zhu Y., Witkowski T., Roche G., Boucheix C., Greco C., Degoul F. (2019). Targeting the Tetraspanins with Monoclonal Antibodies in Oncology: Focus on Tspan8/Co-029. Cancers.

[14] Heo K., Lee S. (2020). TSPAN8 as a Novel Emerging Therapeutic Target in Cancer for Monoclonal Antibody Therapy. Biomolecules.

[15] Zhu Y., Ailane N., Sala-Valdes M., Haghighi-Rad F., Billard M., Nguyen V., Saffroy R., Lemoine A., Rubinstein E., Boucheix C. (2017). Multi-factorial modulation of colorectal carcinoma cells motility—Partial coordination by the tetraspanin Co-029/tspan8. Oncotarget.

[16] Inoue A., Yanagisawa M., Kimura S., Kasuya Y., Miyauchi T., Goto K., Masaki T. (1989). The human endothelin family: Three structurally and pharmacologically distinct isopeptides predicted by three separate genes. Proc. Natl. Acad. Sci. USA.

[17] Remuzzi G., Perico N., Benigni A. (2002). New therapeutics that antagonize endothelin: Promises and frustrations. Nat. Rev. Drug Discov..

[18] Gray G.A., Webb D.J. (1996). The endothelin system and its potential as a therapeutic target in cardiovascular disease. Pharmacol. Ther..

[19] Gadea A., Schinelli S., Gallo V. (2008). Endothelin-1 regulates astrocyte proliferation and reactive gliosis via a JNK/c-Jun signaling pathway. J. Neurosci..

[20] Bagnato A., Rosano L., Spinella F., Di Castro V., Tecce R., Natali P.G. (2004). Endothelin B receptor blockade inhibits dynamics of cell interactions and communications in melanoma cell progression. Cancer Res..

[21] Okazawa M., Shiraki T., Ninomiya H., Kobayashi S., Masaki T. (1998). Endothelin-induced apoptosis of A375 human melanoma cells. J. Biol. Chem..

[22] Pflug B.R., Zheng H., Udan M.S., D’Antonio J.M., Marshall F.F., Brooks J.D., Nelson J.B. (2007). Endothelin-1 promotes cell survival in renal cell carcinoma through the ET(A) receptor. Cancer Lett..

[23] Wright C.E., Fozard J.R. (1988). Regional vasodilation is a prominent feature of the haemodynamic response to endothelin in anaesthetized, spontaneously hypertensive rats. Eur. J. Pharmacol..

[24] Khodorova A., Montmayeur J.P., Strichartz G. (2009). Endothelin receptors and pain. J. Pain.

[25] Smith T.P., Haymond T., Smith S.N., Sweitzer S.M. (2014). Evidence for the endothelin system as an emerging therapeutic target for the treatment of chronic pain. J. Pain Res..

[26] Greco C., Basso L., Desormeaux C., Fournel A., Demuynck B., Lafendi L., Chapiro S., Lemoine A., Zhu Y.Y., Knauf C. (2021). Endothelin-1 Exhibiting Pro-Nociceptive and Pro-Peristaltic Activities Is Increased in Peritoneal Carcinomatosis. Front. Pain Res..

[27] Irani S., Salajegheh A., Smith R.A., Lam A.K. (2014). A review of the profile of endothelin axis in cancer and its management. Crit. Rev. Oncol. Hematol..

[28] Enevoldsen F.C., Sahana J., Wehland M., Grimm D., Infanger M., Kruger M. (2020). Endothelin Receptor Antagonists: Status Quo and Future Perspectives for Targeted Therapy. J. Clin. Med..

[29] Cajot J.F., Sordat I., Silvestre T., Sordat B. (1997). Differential display cloning identifies motility-related protein (MRP1/CD9) as highly expressed in primary compared to metastatic human colon carcinoma cells. Cancer Res..

[30] Zoller M. (2009). Tetraspanins: Push and pull in suppressing and promoting metastasis. Nat. Rev. Cancer.

[31] Ashman L.K., Zoller M., Berditchevski F., Rubinstein E. (2013). Tetraspanins in Cancer. Tetraspanins.

[32] El Kharbili M., Cario M., Bechetoille N., Pain C., Boucheix C., Degoul F., Masse I., Berthier-Vergnes O. (2020). Tspan8 Drives Melanoma Dermal Invasion by Promoting ProMMP-9 Activation and Basement Membrane Proteolysis in a Keratinocyte-Dependent Manner. Cancers.

[33] Voglstaetter M., Thomsen A.R., Nouvel J., Koch A., Jank P., Navarro E.G., Gainey-Schleicher T., Khanduri R., Gross A., Rossner F. (2019). Tspan8 is expressed in breast cancer and regulates E-cadherin/catenin signalling and metastasis accompanied by increased circulating extracellular vesicles. J. Pathol..

[34] Zhu R., Gires O., Zhu L., Liu J., Li J., Yang H., Ju G., Huang J., Ge W., Chen Y. (2019). TSPAN8 promotes cancer cell stemness via activation of sonic Hedgehog signaling. Nat. Commun..

[35] Agaesse G., Barbollat-Boutrand L., Sulpice E., Bhajun R., El Kharbili M., Berthier-Vergnes O., Degoul F., de la Fouchardiere A., Berger E., Voeltzel T. (2017). A large-scale RNAi screen identifies LCMR1 as a critical regulator of Tspan8-mediated melanoma invasion. Oncogene.

[36] Agaesse G., Barbollat-Boutrand L., El Kharbili M., Berthier-Vergnes O., Masse I. (2017). p53 targets TSPAN8 to prevent invasion in melanoma cells. Oncogenesis.

[37] Li J., Chen X., Zhu L., Lao Z., Zhou T., Zang L., Ge W., Jiang M., Xu J., Cao Y. (2021). SOX9 is a critical regulator of TSPAN8-mediated metastasis in pancreatic cancer. Oncogene.

[38] Xu Y., Wu H., Wu L., Xu L., Li J., Wang Q., Pu X. (2020). Silencing of long non-coding RNA SOX21-AS1 inhibits lung adenocarcinoma invasion and migration by impairing TSPAN8 via transcription factor GATA6. Int. J. Biol. Macromol..

[39] Zhang H.S., Liu H.Y., Zhou Z., Sun H.L., Liu M.Y. (2020). TSPAN8 promotes colorectal cancer cell growth and migration in LSD1-dependent manner. Life Sci..

[40] Jackson C.D., Turner A.J. (2002). Detection and assessment of endothelin-converting enzyme activity. Methods Mol. Biol..

[41] Xu D., Emoto N., Giaid A., Slaughter C., Kaw S., deWit D., Yanagisawa M. (1994). ECE-1: A membrane-bound metalloprotease that catalyzes the proteolytic activation of big endothelin-1. Cell.

[42] Schmidt M., Kroger B., Jacob E., Seulberger H., Subkowski T., Otter R., Meyer T., Schmalzing G., Hillen H. (1994). Molecular characterization of human and bovine endothelin converting enzyme (ECE-1). FEBS Lett..

[43] Mzhavia N., Pan H., Che F.Y., Fricker L.D., Devi L.A. (2003). Characterization of endothelin-converting enzyme-2. Implication for a role in the nonclassical processing of regulatory peptides. J. Biol. Chem..

[44] Pacheco-Quinto J., Herdt A., Eckman C.B., Eckman E.A. (2013). Endothelin-converting enzymes and related metalloproteases in Alzheimer’s disease. J. Alzheimers Dis..

[45] Emoto N., Nurhantari Y., Alimsardjono H., Xie J., Yamada T., Yanagisawa M., Matsuo M. (1999). Constitutive lysosomal targeting and degradation of bovine endothelin-converting enzyme-1a mediated by novel signals in its alternatively spliced cytoplasmic tail. J. Biol. Chem..

[46] Awano S., Dawson L.A., Hunter A.R., Turner A.J., Usmani B.A. (2006). Endothelin system in oral squamous carcinoma cells: Specific siRNA targeting of ECE-1 blocks cell proliferation. Int. J. Cancer.

[47] Egidy G., Juillerat-Jeanneret L., Jeannin J.F., Korth P., Bosman F.T., Pinet F. (2000). Modulation of human colon tumor-stromal interactions by the endothelin system. Am. J. Pathol..

[48] Zhao K., Erb U., Hackert T., Zoller M., Yue S. (2018). Corrigendum to “Distorted leukocyte migration, angiogenesis, wound repair and metastasis in Tspan8 and Tspan8/CD151 double knockout mice indicate complementary activities of Tspan8 and CD51” [Biochim. Biophys. Acta 1865(2) (2018) 379–391]. Biochim. Biophys. Acta. Mol. Cell Res..

[49] Lammerding J., Kazarov A.R., Huang H., Lee R.T., Hemler M.E. (2003). Tetraspanin CD151 regulates α6β1 integrin adhesion strengthening. Proc. Natl. Acad. Sci. USA.

[50] Iwamoto R., Higashiyama S., Mitamura T., Taniguchi N., Klagsbrun M., Mekada E. (1994). Heparin-binding EGF-like growth factor, which acts as the diphtheria toxin receptor, forms a complex with membrane protein DRAP27/CD9, which up-regulates functional receptors and diphtheria toxin sensitivity. EMBO J..

[51] Feigelson S.W., Grabovsky V., Shamri R., Levy S., Alon R. (2003). The CD81 tetraspanin facilitates instantaneous leukocyte VLA-4 adhesion strengthening to vascular cell adhesion molecule 1 (VCAM-1) under shear flow. J. Biol. Chem..

