# Supplementary material for: The Tetraspanin Tspan8 Associates with Endothelin Converting Enzyme ECE1 and Regulates Its Activity
Source: Cancers (Basel). 2023 Sep 27;15(19):4751. doi: 10.3390/cancers15194751 (PMC10571763; doi:10.3390/cancers15194751)
Supplement: Supplementary file 1 [file cancers-15-04751-s001.zip › cancers-2584345-supplementary.pdf]

A

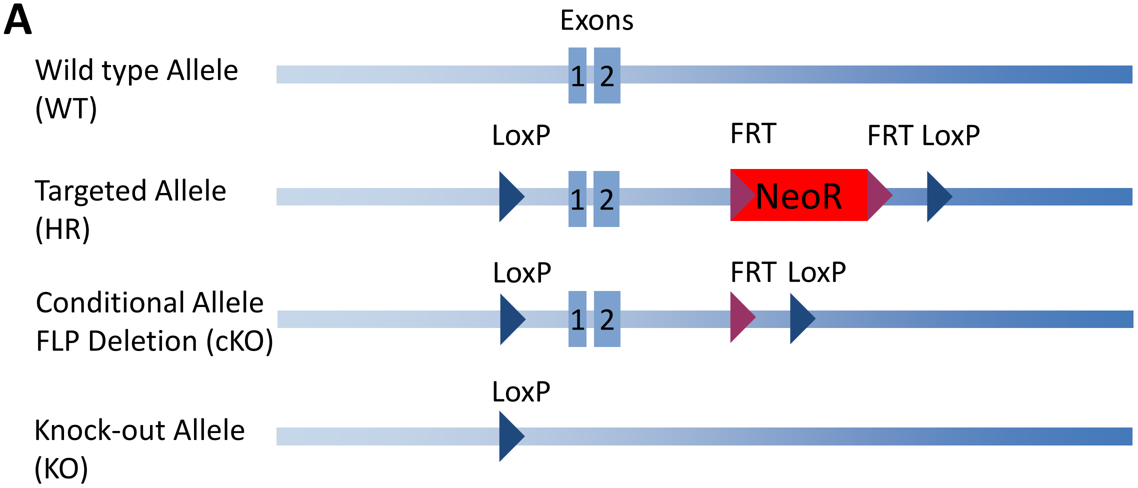

B

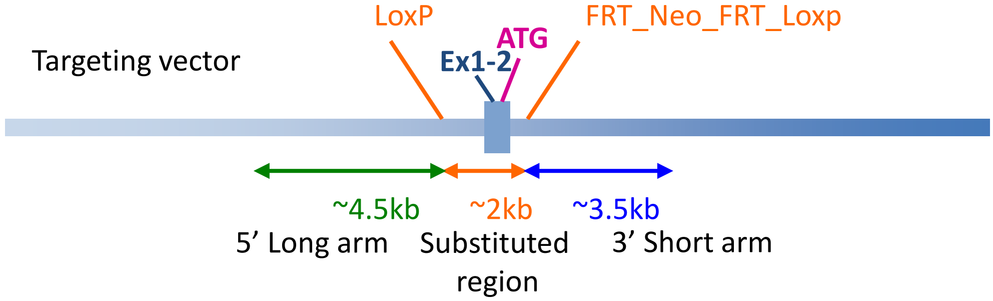

C

Targeting vector sequence

1) 5’ homology long arm (4,5 kb) start 115647448 to 115651966 (NC_000076.7 of GRCm39 assembly)

CGTGAGTTGCTATTACT........CCGACGGTATACGTTGGTG

2) Floxed fragment 1969bp (<u>genomic seq 81-1927, Exon1-2 1299-1678=378bp (E1 66bp, Intron1 141bp, E2 177bp, intron2 252bp</u>)) with *5’lox* and *3’frt* sequences

GGCCGGCCAAGCTTCTCGAGCTTAAGGTCGACCTGCAG*ataacttcgtataatgtatgctatacgaa gttat*TTAATTAA<u>CAGGGCT</u>................<u>TCAAGTTCTCTGA</u>CACCGGTG*gaagttcctattctctagaa agtataggaacttc* **and PGK-Neo^r region 1917bp with *frt* and *lox* sequences**

gcggccaattctaccgggtagg............gaagctcgataccgtcgag*gaagttcctattctctagaaagtataggaa ctt*cgcggccgg*ataacttcgtataatgtatgctatacgaagttat*ggatccatcgaccccctgcag

3) 3’ homology short arm (3,5 kb) end : 115653820 to 11567309 (NC_000076.7 of GRCm39 assembly)

ATAAACATTTCCCGAATGTTTTCATAT............CTAATTGCTTTAATTGGAAACGTATGATCCT TCCTAGGAAATTAGCAATTTGT

Figure S1. Strategy for homologous recombination of the Tspan8 gene

A. A fragment of gene comprising 1.2kb of promoter, Exon1 and 2 (ATG translation initiation site is in Exon2) and a short Intron 2 sequence is replaced by the same floxed sequence containing a resistance gene flanked by FRT sites. Removal of the resistance gene generates a conditional knockout sequence. Removal of the LoxP flanked sequence generates the gene knockout.

B. A vector was built comprising 5’ and 3’ homology arms around the floxed region

C. Sequences of the plasmid construct. Start and end numbers refer to Genome Data Viewer <u>http://https://www.ncbi.nlm.nih.gov/genome/gdv/ (NC_000076.7 of GRCm39 assembly)</u>. Natural genomic sequences are indicated in bold. FRT and LoxP sequences are in green and pink small italic characters.
